# Ablation of endothelial prolyl hydroxylase domain protein‐2 promotes renal vascular remodelling and fibrosis in mice

**DOI:** 10.1111/jcmm.13117

**Published:** 2017-03-07

**Authors:** Shuo Wang, Heng Zeng, Sean T. Chen, Liying Zhou, Xue‐Jiao Xie, Xiaochen He, Yong‐Kang Tao, Qin‐hui Tuo, Changqin Deng, Duan‐fang Liao, Jian‐Xiong Chen

**Affiliations:** ^1^ Department of Pharmacology and Toxicology University of Mississippi Medical Center Jackson MS USA; ^2^ Duke University School of Medicine Durham NC USA; ^3^ Hunan University of Chinese Medicine Changsha Hunan China

**Keywords:** PHD2, HIF, Endothelium, renal fibrosis, Pericyte coverage

## Abstract

Accumulating evidence demonstrates that hypoxia‐inducible factor (HIF‐α) hydroxylase system has a critical role in vascular remodelling. Using an endothelial‐specific prolyl hydroxylase domain protein‐2 (PHD2) knockout (PHD2^EC^KO) mouse model, this study investigates the regulatory role of endothelial HIF‐α hydroxylase system in the development of renal fibrosis. Knockout of PHD2 in EC up‐regulated the expression of HIF‐1α and HIF‐2α, resulting in a significant decline of renal function as evidenced by elevated levels of serum creatinine. Deletion of PHD2 increased the expression of Notch3 and transforming growth factor (TGF‐β1) in EC, thus further causing glomerular arteriolar remodelling with an increased pericyte and pericyte coverage. This was accompanied by a significant elevation of renal resistive index (RI). Moreover, knockout of PHD2 in EC up‐regulated the expression of fibroblast‐specific protein‐1 (FSP‐1) and increased interstitial fibrosis in the kidney. These alterations were strongly associated with up‐regulation of Notch3 and TGF‐β1. We concluded that the expression of PHD2 in endothelial cells plays a critical role in renal fibrosis and vascular remodelling in adult mice. Furthermore, these changes were strongly associated with up‐regulation of Notch3/TGF‐β1 signalling and excessive pericyte coverage.

## Introduction

Pathological fibrosis in vital organs causes significant morbidity and mortality in both developing and developed countries. An estimated 13% of the general population has some degree of chronic kidney disease (CKD) 1, and 45% of them dies of fibrotic‐associated disorders 2. The progression of CKD is a consequence of destructive fibrosis, commonly secondary to glomerulonephritis, diabetes mellitus, obstructive nephropathy, interstitial nephritis and cystic nephropathies 3. Renal fibrosis, characterized by tubule‐interstitial fibrosis and glomerulosclerosis, disrupts the normal structure of the kidney and ultimately leads to irreversible decline of renal function. The hallmark of renal fibrosis is characterized by activation of fibroblasts and deposition of abundant extracellular matrix (ECM). Fibroblasts, which can transdifferentiate into myofibroblasts, are mesenchymal cells embedded in the ECM of connective tissues of organs. It secretes a particular molecular collagen that forms the extracellular fibrillary matrix 4. To date, the exact origin of renal myofibroblasts is still debated in the CKD. Accumulating evidence has converged on the concept that pericytes, resident interstitial mesenchymal cells supporting capillary endothelium, are the main source of myofibroblasts in renal fibrosis. Studies have shown that interstitial mesenchymal cells could be differentiated into myofibroblasts in rat models for obstructive nephropathy 5. In the Ang‐II‐induced renal fibrosis model, the majority of myofibroblasts was derived from resident renal interstitial cells, which were later identified as perivascular pericytes 6, 7. Using pericyte lineage tracing reporter mice, a recent study further substantiated the notion that pericytes are accountable for the majority of myofibroblasts in the renal fibrosis 8.

Prolyl hydroxylase domain proteins (PHD) are oxygen‐sensing molecules that degrade hypoxia‐inducible factor‐α (HIF‐α) 9, 10, 11, 12. Of the three PHD isoforms, PHD2 is considered the most important HIF‐α‐regulating variant 13, 14, 15. Recent studies have shown that inactivation of PHD2 in endothelial cells (EC) causes profound vascular remodelling of peripheral pulmonary arteries and leads to pulmonary arterial hypertension 16, 17. Our recent study indicates that knockout of PHD2 increases EC/pericyte coverage *via* activation of HIF‐2α/Notch3 signalling pathway in the lung of LPS‐treated mice 18. To date, no studies have investigated the regulatory role of endothelial PHD2 in the development of renal fibrosis.

In this study, we suggested that ablation of PHD2 in EC promotes renal arteriolar remodelling and results in fibrosis by increasing pericyte differentiation into myofibroblasts. Using an endothelial‐specific PHD2 knockout (PHD2^EC^KO) mouse, the contribution of endothelial PHD2 in the development of renal fibrosis was investigated. We found that PHD2^EC^KO mice developed renal fibrosis, and this pathology was strongly associated with the remodelling of arterioles and pericyte differentiation into myofibroblasts. Mechanistically, we found that these alternations may be associated with up‐regulation of Notch3 and TGF‐β1 expression in the kidney.

## Materials and methods

All procedures conformed to the Institute for Laboratory Animal Research Guide for the Care and Use of Laboratory Animals and were approved by the University of Mississippi Medical Center Animal Care and Use Committee (Protocol ID: 1280A). The investigation conforms to the Guide for the Care and Use of Laboratory Animals published by the US National Institutes of Health (NIH Publication No. 85‐23, revised 1996).

### Generation of the PHD2^flox/flox^ ^(f/f)^ and PHD2^ECKO^ mice

PHD2^flox/flox^ (PHD2^f/f^) mice were obtained from Dr. Guo‐hua Fong at University of Connecticut. PHD2^EC^KO mice were generated using the Cre‐LoxP system as we previously reported 19. In brief, exon 2 of PHD2 gene in PHD2^f/f^ mice was flanked with LoxP sites, for subsequent deletion by Cre recombinase. PHD2^f/f^ mice were crossbred with VE‐Cadherin‐Cre (Cdh5‐Cre) transgenic mice [B6.FVB‐Tg (Cdh5‐cre) 7Mlia/J] obtained from Jackson Laboratories that express Cre recombinase under the control of Cdh5 promoter in vascular ECs. The resulting Cdh5‐Cre/PHD2^flox/−^ heterozygous mutants were then mated with PHD2^f/f^ to obtain endothelial‐specific ablated PHD2 mutant mice (PHD2^EC^KO) and PHD2^f/f^ mice. Experiments were performed on male mice at 15 months of age.

### Measurement of blood pressure

Systemic blood pressure was monitored in the training mice by tail‐cuff occlusion method, according to the manufacturer's instructions (Hatteras Instruments, Cary, NC, USA). In brief, the measurements were continued every day for 1 week. The first 3 days were used as an adjustment period for the mice and not included in the results, and measurements for remaining days were averaged for the final results. This method of blood pressure analysis has been validated extensively 20.

### Measurement of serum creatinine levels

At the end of experiments, animals were killed and chest was opened. Blood (1 ml) was collected from the heart. The levels of creatinine were measured by a colorimetric assay (Lab‐assay™ creatinine kit; Wako Pure Industrial Ltd, Osaka, Japan). In brief, the samples (50 μl) were deproteinized with sodium tungstate and phosphoric acid (300 μl). The supernatant was separated. Creatinine in the samples was combined with picric acid in alkaline solution to produce tangerine condensate (Jaffe reaction). Quantitation of total creatinine in the sample was calculated by measurement of the absorbance at 520 nm.

### Western blot analysis

Mouse renal cortex tissues were homogenized in 300 μl of an ice‐cold lysis buffer. The homogenates were centrifuged at 16000 × g for 15 min. at 4°C, and the total protein concentrations were determined using a BCA protein assay kit (Pierce Co, Rockford, IL, USA). An aliquot (30 μg) of the protein lysate was separated on a 10% SDS‐PAGE gel and transferred to a polyvinylidene difluoride membrane by electrophoresis. The membranes were blocked with 5% nonfat dry milk in Tris‐buffered saline and incubated with the following primary antibodies overnight: neural glial antigen (NG) 2, FSP‐1, apelin (1:1000; Abcam, Cambridge, MA, USA), PHD2, HIF‐1α and HIF‐2α (1:1000; Novus Bio, Littleton, CO, USA), Notch3, TGF‐β1, angiopoietin‐1 (Ang‐1) and angiopoietin‐2 (Ang‐2) (1:1000; Sigma‐Aldrich, St. Louis, MO, USA), apelin receptor (APJ), vascular endothelial growth factor (VEGF) (1:1000; Santa Cruz, CA, USA) and VEGF receptor 2 (VEGFR2) (1:1000; Cell Signaling, Danvers, MA, USA). The membranes were then washed and incubated for 2 hrs with an anti‐rabbit or antimouse secondary antibody conjugated with horseradish peroxidase (1:5000; Santa Cruz). Densitometric analyses of the bands were carried out using image acquisition and analysis software (TINA 2.0).

### Histological and immunofluorescence analysis

The renal cortex tissues were fixed with buffered 10% formalin solution (SF93–20; Fisher Scientific, Pittsburgh, PA, USA), embedded in frozen OCT compound (4583; Sakura Finetek, Torrance, CA, USA) and 10 μm frozen sections prepared. Some sections were stained with haematoxylin & eosin. Some were directly immunostained with Alexa 488‐labelled Isolectin B4 (IB4) for ECs, smooth muscle actin (SMA) and a NG2 antibody for pericytes (1:100; Abcam). Other sections were immunostained with Notch3, PHD2, FSP‐1 and TGF‐β1 primary antibodies (1:200) followed by incubation with second antibodies conjugated with fluorescein isothiocyanate (FITC) or Cy3 (1:500). Photomicrographs were obtained with an Olympus BX51 microscope, a Q‐Color5 digital camera and a Q‐Capture Suite acquisition software (Olympus, Tokyo, Japan). The area percentage of fluorescence intensity was quantified at six random microscopic fields using image analysis software (Image J, NIH, Bethesda, MD, USA). Masson trichrome staining was also performed to measure the degree of fibrosis (blue) in the renal cortex.

### Glomerular injury score

Approximately 80 glomeruli from each kidney were examined for glomerular injury score using the sections stained with Masson trichrome. Each glomerulus was graded from 1 to 4 by the method previously described 21. In brief, each glomerulus from a kidney slice was scored from 0 (no injury) to 4 (nonfunctional, sclerotized). Each score was then calculated according to the formula for Glomerular Injury Score = [(1 × number of grade 2 glomeruli)+(2 × number of grade 3 glomeruli)+(3 × number of grade 4 glomeruli)] × 100/(number of glomeruli observed) 21.

### Ultrasonography for measurement of renal RI

Mice were anaesthetized by inhaled isoflurane and immobilized on a heating platform to maintain body temperature at 37°C. Heart rate was monitored by electrocardiogram (ECG) electrode. Peak systolic and end‐diastolic renal arterial blood flow velocities were measured using pulsed‐wave Doppler. Renal RI was calculated using the equation as following: RI = (peak systolic velocity‐end‐diastolic velocity)/peak systolic velocity.

### Statistical methods

Data are presented as mean ± S.E.M. The significance of differences in the means of corresponding values between groups was determined using the unpaired Student's *t*‐test. A *P* < 0.05 was considered to be significant.

## Results

### The phenotype of the PHD2^EC^KO mice

**Figure 1:**
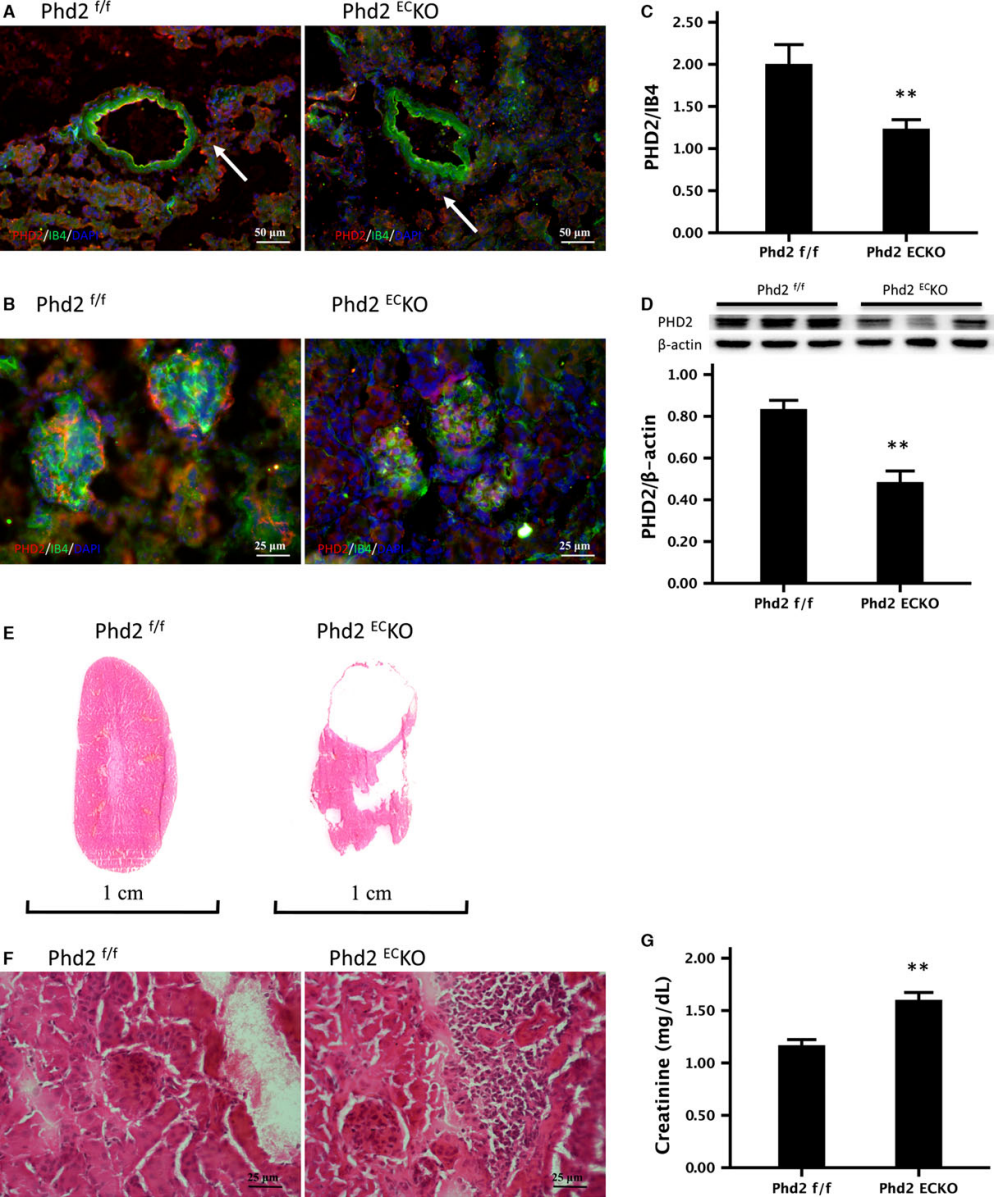
The renal phenotype of PHD2^f/f^ and PHD2^EC^KO mice. (**A**) In the PHD2^EC^KO mice, PHD2‐positive cells were only seen in the perivascular region (white arrows), but not presented in the endothelium of small arteries. (**B**) In the PHD2^f/f^ mice, PHD2‐positive cells were observed in the glomerular capillary (yellow), but not found in the PHD2^EC^KO mice. (**C**) The area percentage of fluorescence intensity of PHD2 in the kidney of PHD2^EC^KO mice decreased significantly (*n* = 9 mice/group). (**D**) Western blot analysis indicated there was a significant decline in the expression of PHD2 in the kidney of PHD2^EC^KO mice (*n* = 6–7 mice). (**E**) The PHD2^EC^KO mice had developed a polycystic‐like phenotype in kidney with a reduced size. (**F**) The glomerulus atrophy and the myofibroblasts accumulating around the arterioles were seen in the PHD2^EC^KO mice. (**G**) The serum creatinine level in the PHD2^EC^KO mice was significantly increased compared to the control PHD2^f/f^ mice (*P* = 0.001) (*n* = 5–7 mice). Mean ± S.E.M., **P < 0.01.

Histological studies were performed to examine the renal phenotype of endothelial PHD2 deficiency mice. First, the renal cortex sections were co‐stained with PHD2 (red) and endothelial marker IB4 (green) to validate deletion of PHD2 in the EC. In the PHD2^EC^KO mice, PHD2‐positive cells were only seen in the perivascular region, but not in the endothelium of small arteries (Fig. 1A) and capillary of glomerulus (Fig. 1B). In the control PHD2^f/f^ mice, PHD2‐positive cells were observed in the renal vascular endothelium (yellow). Both the fluorescent intensity measurement and Western blot analysis indicated there was a significant reduction in PHD2 expression in the kidney of PHD2^EC^KO mice (Fig. 1C and D). The PHD2^EC^KO mice developed a polycystic phenotype with a small kidney (Fig. 1E). Glomerular atrophy and the accumulating myofibroblasts around the arterioles were observed (Fig. 1F). The levels of serum creatinine were significantly increased (Fig. 1G), suggesting an impairment of renal function in the PHD2^EC^KO mice.

As shown in Table 1, the heart rate and mean arterial pressure (MAP) were significantly increased in the PHD2^EC^KO mice compared to control PHD2^f/f^ mice. Moreover, the PHD2^EC^KO mice had a significantly higher diastolic blood pressure than the control PHD2^f/f^ mice (Table 1).

**Table 1:** Systemic arterial blood pressure

	PHD2^f/f^	PHD2^EC^KO	*t*	*P*
HR (bpm)	618.6 ± 19.6	720.6 ± 13.1	−4.415	0.001
SBP (mmHg)	108.0 ± 1.3	114.9 ± 2.6	−2.247	0.040
DBP (mmHg)	86.3 ± 2.3	95.3 ± 2.4	−2.695	0.017
MAP (mmHg)	93.5 ± 1.6	101.9 ± 2.4	−2.796	0.014
Pulse pressure (mmHg)	21.8 ± 2.5	19.6 ± 1.4	0.775	0.451

HR, heart rate; SBP, systolic blood pressure; DBP, diastolic blood pressure; MAP, mean arterial pressure.

### Renal arterial remodelling in the PHD2^EC^KO mice

**Figure 2:**
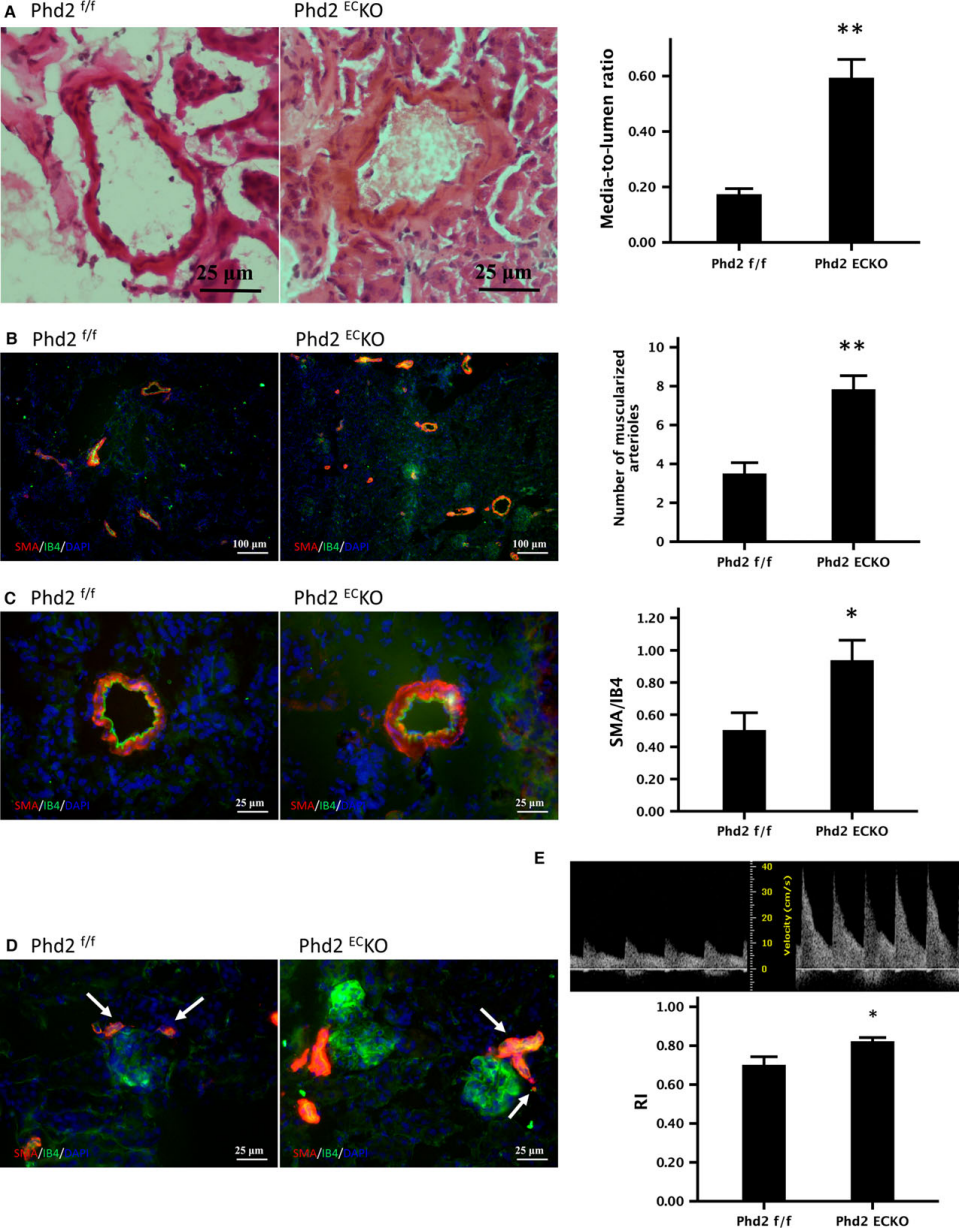
Deletion of PHD2 in EC causes renal arterial remodelling in mice. (**A**) Haematoxylin & eosin staining indicated that the media‐to‐lumen ratio of small arteries <1000 μm was significantly increased (*n* = 6 mice/group). (**B**) Immuno‐histo‐staining labelled VSMC with SMA (red) and endothelial cells with IB4 (green) showed the number of muscularized arterioles increased significantly in the PHD2^EC^KO mice (*n* = 6 mice/group). (**C**) The area of SMA/IB4 of the renal arteries with similar diameter was significantly increased in the PHD2^EC^KO mice (*n* = 9 mice/group). (**D**) High‐magnification images indicated a proliferation of smooth muscles in the afferent and efferent glomerular arterioles (white arrows). (**E**) Presentative image of peak systolic and end‐diastolic renal arterial blood flow velocity measured by pulsed‐wave Doppler. Renal RI was calculated. RI was significantly increased in the PHD2^EC^KO mice compared to the control PHD2^f/f^ mice (*n* = 6 mice/group). Mean ± S.E.M., *P < 0.05, **P < 0.01.

Haematoxylin and Eosin staining indicated arterial remodelling in the renal cortex of PHD2^EC^KO mice, especially in the small artery. The media‐to‐lumen ratio was significantly increased in the kidney of PHD2^EC^KO mice (Fig. 2A). Kidney sections were further double labelled with EC marker IB4 and vascular smooth muscle cell marker α‐SMA to assess the renal arterial remodelling. As shown in Figure 2B, the number of muscularized arterioles increased significantly in the PHD2^EC^KO mice. The area of SMA/IB4 with similar diameter was further calculated in the kidney. As shown in Figure 2C, the area of SMA/IB4 fluorescent intensity was significantly increased in the PHD2^EC^KO mice. High‐magnification images further confirmed that increased smooth muscle cells were in the afferent and efferent glomerular arterioles (Fig. 2D). Using ultrasonography, we examined the renal RI, a ratio of peak systolic and end‐diastolic velocity differences across the renal artery. RI has been used as an indirect measurement of renal blood flow and vascular resistance. Renal vascular remodelling affects the resistance vessels in the kidney to increase RI. In PHD2^EC^KO mice, the value of RI was significantly increased compared to control PHD2^f/f^ mice (Fig. 2E).

### The expression of HIF‐α and pericyte recruiting genes

**Figure 3:**
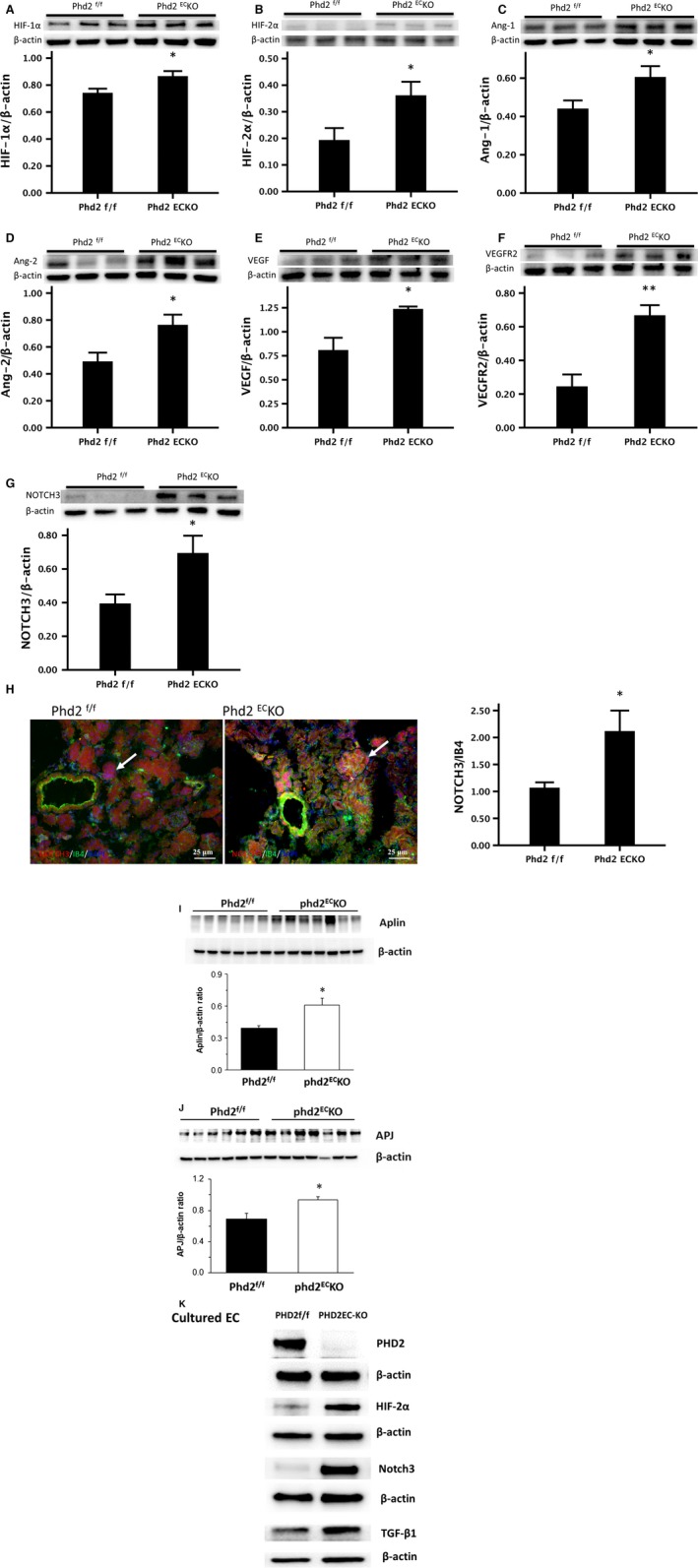
The expression of pericyte‐associated factors in the kidney. (**A‐G**) Western blot showed that the expression of HIF‐1α, HIF‐2α, Ang‐1, Ang‐2, VEGF, VEGFR2 and NOTCH3 was significantly increased in the renal cortex of PHD2^EC^KO mice compared to those of the control PHD2^f/f^ mice (*n* = 6–7 mice). (**H**) The immunofluorescence study further confirmed that NOTCH3 (red) was up‐regulated in the kidney of PHD2^EC^KO mice compared to the control mice especially around the vessel and in the glomerulus (white arrows) (*n* = 9 mice/group). Mean ± S.E.M., *P < 0.05, **P < 0.01. (**I** and **J**) Western blot showed that the expression of apelin and apelin receptor (AJP) was significantly increased in the renal cortex of PHD2^EC^KO mice compared to those of the control PHD2^f/f^ mice (*n* = 6–7 mice). (**K**) The expression of HIF‐2α and Notch3/TGFβ in the cultured EC isolated from PHD2^EC^KO mice and control PHD2^f/f^ mice.

PHD2 deficiency in EC significantly increased the expression of HIF‐1α and HIF‐2α in the kidney (Fig. 3A and B). This was accompanied by a significant increase in the pericyte target genes Ang‐1 and Ang‐2 expression (Fig. 3C and D). Similarly, the expression of VEGF and VEGFR2 was significantly increased (Fig. 3E and F). Notch3 has been implicated in controlling pericyte proliferation and differentiation 22. The expression of Notch3 was significantly up‐regulated in the kidney of PHD2^EC^KO mice (Fig. 3G). The fluorescence intensity measurement study further confirmed that NOTCH3 (red) was increased in the renal cortex. This was especially prominent around the vessels and the glomeruli (Fig. 3H), suggesting increased NOTCH3 may contribute to the pericyte differentiation in the PDH2^EC^ KO mice. In addition, the expression of apelin and AJP was significantly increased in the PDH2^EC^ KO mice (Fig. 3I and J). To further test whether endothelial PHD2 contributed to the up‐regulation of these protein expressions, ECs were isolated from PHD2^f/f^ and PHD2^EC^ KO mice. As shown in Figure 3K, the expression of HIF‐2, Notch3, and TGF‐β1 was dramatic increased in the cultured EC of PDH2^EC^ KO mice.

### Increased pericyte coverage in the renal vasculature of PHD2^EC^KO mice

**Figure 4:**
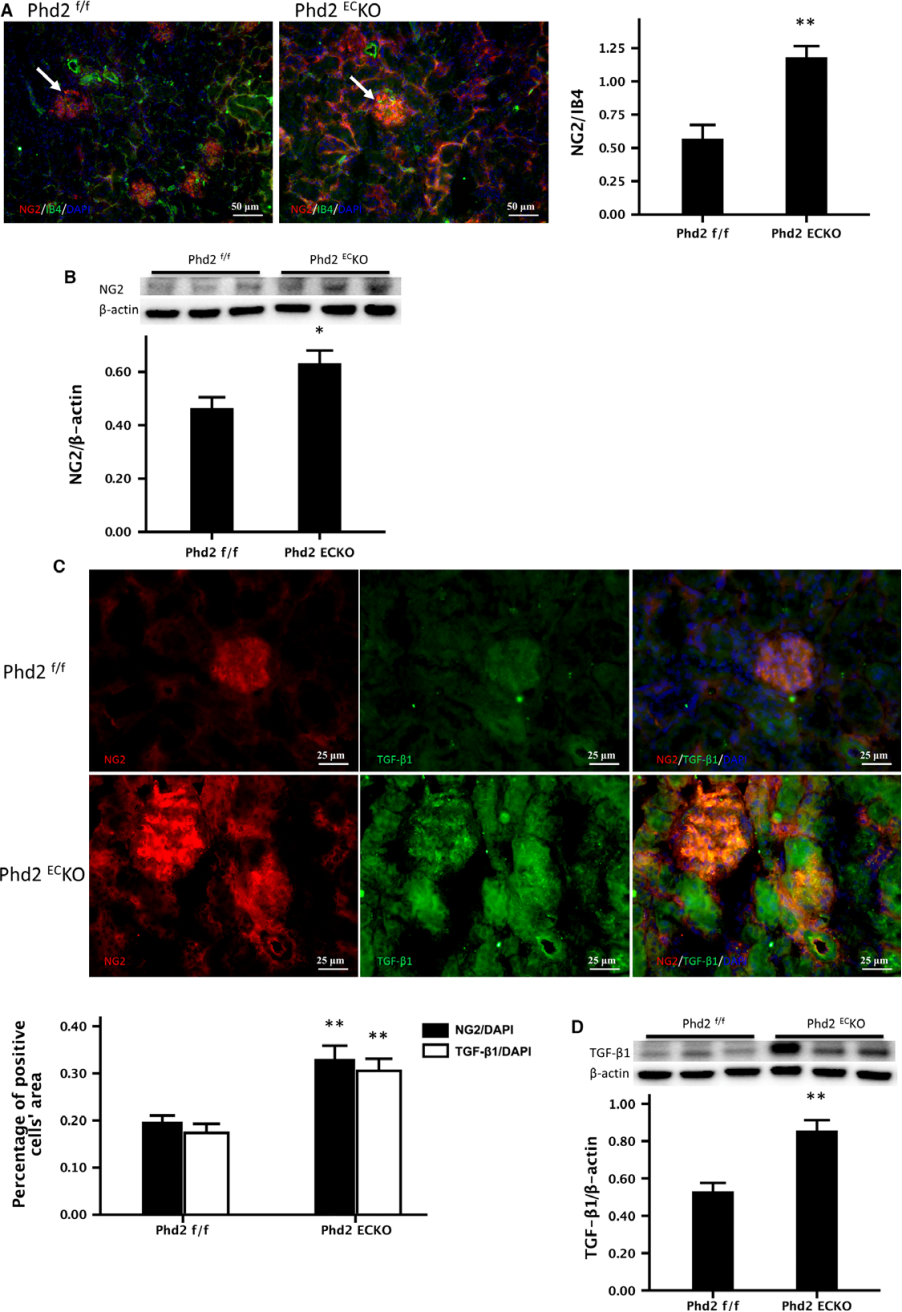
The recruitment of pericyte in the renal arterioles and glomeruli. (**A**) There were more NG2‐positive cells around the arterioles and increased pericyte coverage (NG2/IB4) in the glomerulus (white arrows) compared to PHD2^f/f^ mice. The area of NG2/IB4 was significantly increased (*n* = 9 mice/group). (**B**) Western blot analysis indicated a significant increase in the expression of NG2 in the kidney of PHD2^EC^KO mice (*n* = 6–7 mice). (**C**) More TGF‐β1^+^ and TGF‐β1^+^/NG2^+^ double‐positive cells (yellow) were found in the kidney of PHD2^EC^KO mice, especially in the glomeruli. Both the areas of NG2/DAPI and TGF‐β1/DAPI were increased significantly (*n* = 9 mice/group). (**D**) Western blot showed that the expression of TGF‐β1 was significantly increased in the kidney of PHD2^EC^KO mice (*n* = 6–7 mice). Mean ± S.E.M., *P < 0.05, **P < 0.01.

In the PHD2^EC^KO mice, fluorescent intensity measurement showed that there were more NG2‐positive cells around the arterioles and increased pericyte coverage (NG2/IB4) in the glomerulus compared to that of the PHD2^f/f^ mice (Fig. 4A). Western blot analysis also indicated a significant increase in the expression of NG2 in the kidney of PHD2^EC^KO mice (Fig. 4B).

TGF‐β1 has been reported to promote pericyte recruitment and pericyte coverage in pulmonary hypertension 23. Consistent with this study, our immunohistochemistry analysis showed that the number of TGF‐β1^+^/NG2^+^ double‐positive cells (yellow) were significantly increased, especially in the glomeruli of the PHD2^EC^KO mice (Fig. 4C). Similarly, the expression of TGF‐β1 was significantly increased (Fig. 4D), suggesting an involvement of TGF‐β1 signalling pathway in the pericyte recruitment and increased coverage of pericytes in the PHD2^EC^KO mice.

### Increased perivascular and renal fibrosis in the PHD2^EC^KO mice

**Figure 5:**
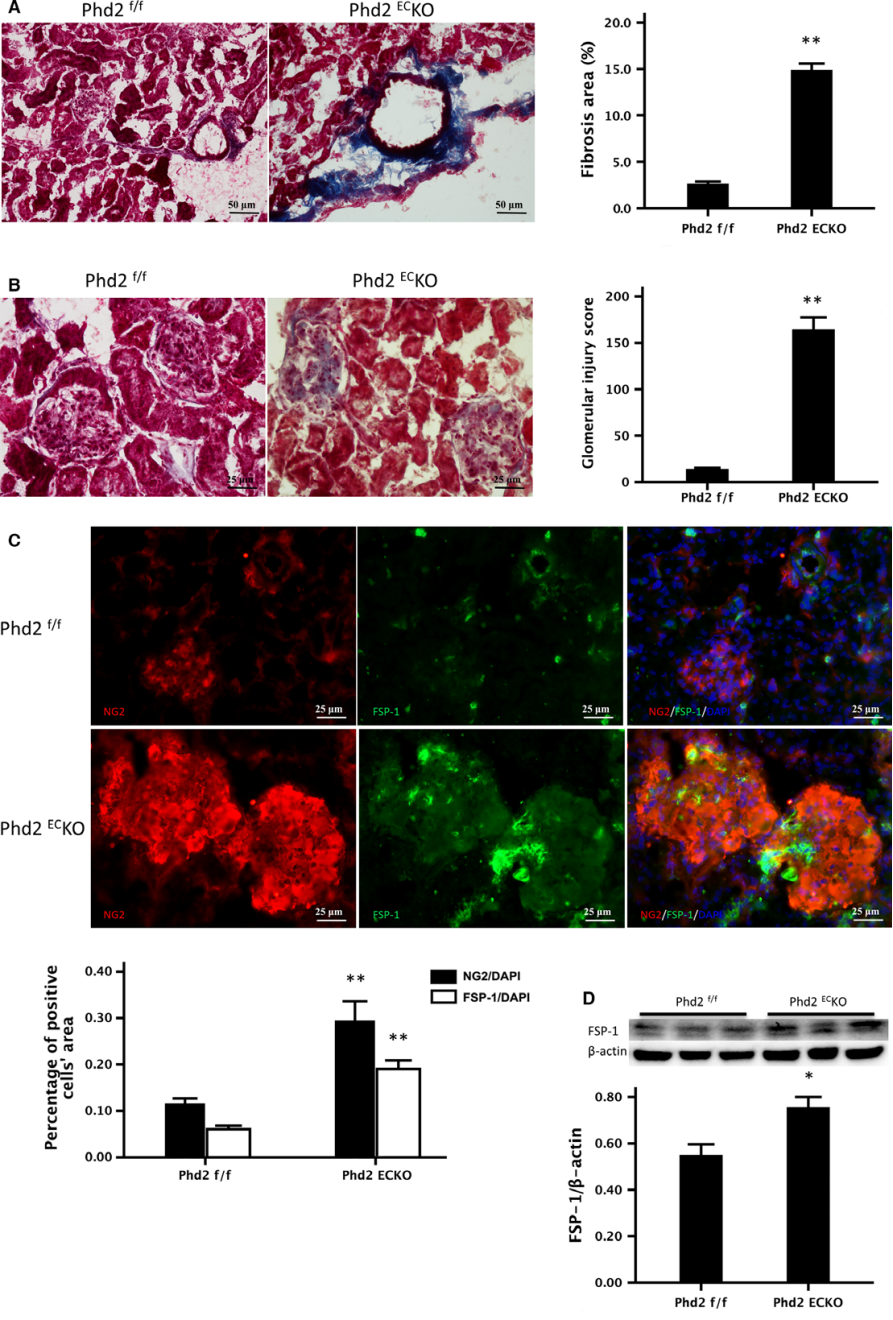
The Relationship between the pericytes and renal fibrosis. (**A**) Masson trichrome staining showed that there was significantly increase in fibrosis formation (blue) in the small renal arteries of PHD2^EC^KO mice. The areas of fibrosis around the arteries in the PHD2^EC^KO mice were significantly increased compared with the control. (*n* = 5 mice/group). (**B**) Representative images of glomeruli in the trichrome‐stained kidney. Glomerular sclerosis and Bowman's capsule fibrosis were increased in the PHD2^EC^KO mice. Average glomerular injury score was significantly higher in the kidneys of PHD2^EC^KO mice compared to that of the control PHD2^f/f^ mice. (**C**) In the PHD2^EC^KO mice, both pericyte marker NG2 (red) and myofibroblast marker FSP‐1 (green) labelled cells were increased and scattered in the glomeruli, especially in the mesangium. Both the areas of NG2/DAPI and FSP‐1/DAPI measured by immunofluorescence were increased significantly in the PHD2^EC^KO mice compared to that of the control PHD2^f/f^ mice (*n* = 9 mice/group). (**D**) Western blot analysis showed that there was a significant increase in expression of FSP‐1 in the kidney of PHD2^EC^KO mice (*n* = 6–7 mice). Mean ± S.E.M., *P < 0.05, **P < 0.01.

There was a significant increase in perivascular fibrosis of renal arterioles in PHD2^EC^KO mice compared to age‐matched control PHD2^f/f^ littermates. The area of fibrosis around the arterioles in PHD2^EC^KO mice was significantly increased compared to control PHD2^f/f^ mice (Fig. 5A, blue). As shown in Figure 5B, glomerular sclerosis and Bowman's capsule fibrosis were found in PHD2^EC^KO mice. Glomerular injury score was significantly increased in the PHD2^EC^KO mice compared to the PHD2^f/f^ mice. To determine whether pericytes transdifferentiated into myofibroblasts, colocalization was analysed by a double stain with NG2 (red) and FSP‐1 (green), a marker of myofibroblasts, in the glomeruli. As shown in Figure 5C, the number of NG2^+^/FSP‐1^+^ was significantly increased and scattered in the glomeruli, especially in the mesangium, indicating that glomerular fibrosis might be due to the differentiation of pericytes into myofibroblasts (Fig. 5C). Western blot analysis further confirmed increased expression of FSP‐1 in the renal cortex of PHD2^EC^KO mice (Fig. 5D).

## Discussion

In the present study, we found that PHD2 deficiency in the endothelium causes renal fibrosis and impaired renal function in mice. Furthermore, PHD2 deficiency led to excessive pericyte coverage and increased media‐to‐lumen ratio in renal arterioles. These alterations resulted in increased renal vessel resistance, which may reduce renal perfusion and cause glomerular hypoxia/ischaemia, ultimately leading to glomerular injury. Mechanistically, we found that loss of PHD2 in EC up‐regulated Notch3 and TGF‐β1 levels, which may enhance pericyte recruitment and differentiation into myofibroblasts. Our results suggest an important regulatory role of endothelial PHD2 in renal vascular remodelling and fibrosis.

Pericyte differentiation into myofibroblasts is considered to be a hallmark of CKD 24, 25, 26. Pericytes are vascular mural cells, which attach to the ECs of capillaries (*e.g*. glomerular capillaries), precapillary arterioles, post‐capillary venules and collecting venules 27. When cells of mesenchymal origin do not make contact with ECs or move away, they are known as ‘perivascular fibroblasts’ or ‘adventitial fibroblasts’ 28, 29, 30. Accumulating evidence suggests that increased pericyte proliferation has a fundamental role in the pathological processes of fibrosis, inflammation, thrombosis and vessel calcification 31, 32. Studies have shown that pericytes transdifferentiate into myofibroblasts under profibrotic stimulations 33, 34, 35. Recent studies have also demonstrated endothelial HIF hydroxylase system plays a critical role in vascular remodelling 16, 17. However, the direct roles of endothelial PHD2 on pericyte and renal fibrosis have not yet been investigated. In the present study, we found the deletion of PHD2 induced excessive pericyte coverage and perivascular fibrosis. The expression of NG2 and FSP‐1 was significantly increased in the kidney of PHD2^EC^KO mice, and specifically, we found that pericytes with NG2^+^/FSP‐1^+^ were centred on the glomerular arteries. These data suggest that pericytes may differentiate into myofibroblasts, resulting in renal fibrosis and impaired renal function. Our next questions are why and how loss of PHD2 in EC causes these alterations. TGF‐β1 is considered the key mediator of renal fibrosis in the CKD 36. The primary role of TGF‐β1 on ECs is to induce EC differentiation into myofibroblasts 37, 38. TGF‐β1 has been reported to be produced by resident renal cells and infiltrating leucocytes during proteinuria 39. In this study, we found that both the number of NG2^+^/TGF‐β1^+^ cells and the expression of TGF‐β1 were significantly increased in the PHD2^EC^KO mice. Moreover, the expression of TGF‐β1 in cultured EC isolated from PHD2^EC^KO mice was increased. These data provide strong evidence that activation of TGF‐β1 signalling pathway in EC may contribute to pericyte differentiation into myofibroblasts.

Arterial remodelling was found extensively in the PHD2^EC^KO mice, which was characterized by structural remodelling of small arterioles, thickening of the vessel wall, and luminal occlusion. The media‐to‐lumen ratio of the renal artery was significantly increased, and most importantly, renal RI was elevated. Therefore, we speculate that increased perivascular fibrosis might reduce the compliance of the renal artery and increase arterial resistance. In turn, this will reduce glomerular perfusion, and ultimately cause glomerular hypoxia/ischaemia and sclerosis. Furthermore, increased pericyte coverage has been shown to contribute to pulmonary hypertension 23. However, the underlying mechanisms that cause excessive pericyte coverage remain undefined. NOTCH3 has been shown to regulate pericyte number and pericyte/EC coverage 40, 41. Notch3 has also been shown to play crucial roles in the human pulmonary arterial remodelling and pulmonary hypertension 22. However, little is known about NOTCH3 on the pericyte density in arterial remodelling. Our recent study suggests that endothelial PHD2 may regulate lung microvascular pericyte/EC coverage and induce pulmonary hypertension *via* up‐regulation of Notch3 19. In line with this, we found that specific deletion of PHD2 in EC up‐regulated NOTCH3 in the renal arteries. These results suggest the importance of NOTCH3 in endothelial PHD2 inactivation‐induced excessive pericyte coverage. In addition, the expression of Ang‐1 was up‐regulated in the PHD2^EC^KO mice. Ang‐1 plays a critical role in the regulation of pericyte recruitment. Previously, we have shown that overexpression of Ang‐1 increases VSMC recruitment *via* NOTCH3 42. Intriguingly, Ang‐1 levels were also elevated in the kidney of PHD2^EC^KO mice. Ang‐1 is essential for pericyte recruitment. Taken together, our present study indicates that activation of Ang‐1/Notch3 signalling pathway may be involved in reciprocal communication between ECs and pericytes, leading to excessive pericyte coverage as well as pericyte differentiation into myofibroblasts. PHD2^EC^KO mice have developed pulmonary hypertension with a right ventricular dysfunction. It is unknown whether these abnormalities contribute to renal fibrosis and dysfunction. In addition, the present study did not address whether young mice develop similar renal phenotype as aged PHD2^EC^KO mice. Therefore, the cardio‐renal phenotype and young mice warrant further investigation in the PHD2^EC^KO mice.

Our study also revealed increased expression of VEGF and VEGFR2 after endothelial PHD2 deletion. VEGF/VEGFR2 signalling is crucial for maintenance of endothelial–pericyte interactions 43, 44, 45. Blocked VEGFR2 on ECs or blocked PDGFR‐b on pericytes resulted in the pericyte detachment after renal injury 46. As a downstream target gene of HIF‐α, it was not surprising that VEGF/VEGFR2 was enhanced by endothelial PHD2 deletion.

In summary, our current work demonstrated a critical role of endothelial PHD2 in the renal fibrosis. Deletion of PHD2 in the microvascular endothelium increased pericyte coverage and enhanced vascular remodelling. Mechanistically, this process may be driven by the up‐regulation of the NOTCH3/TGF‐β1 pathways.

## Sources of funding

This study was supported by grants from NIH grant 2R01HL102042‐05 and University of Mississippi Medical Center Intramural Research Support Program to J.X. Chen.

## Conflict of interest

There are no conflict of interests.
